# Evaluating the levels of interleukin-1 family cytokines in sporadic amyotrophic lateral sclerosis

**DOI:** 10.1186/1742-2094-11-94

**Published:** 2014-05-23

**Authors:** Paola Italiani, Cecilia Carlesi, Paola Giungato, Ilaria Puxeddu, Barbara Borroni, Paola Bossù, Paola Migliorini, Gabriele Siciliano, Diana Boraschi

**Affiliations:** 1 Laboratory of Innate Immunity and Cytokines, Institute of Biomedical Technologies, National Research Council, Via G. Moruzzi 1, 56124 Pisa, Italy; 2 Department of Clinical and Experimental Medicine, Neurology Unit, University of Pisa, Via Roma 55, 56126 Pisa, Italy; 3 Department of Clinical and Experimental Medicine, Clinical Immunology Unit, University of Pisa, Via Roma 55, 56126 Pisa, Italy; 4 Neurology Unit, University of Brescia, P.le Spedali Civili 1, 25123 Brescia, Italy; 5 Laboratory of Experimental Neuropsychobiology, IRCCS Fondazione Santa Lucia, Via Ardeatina 306, 00179 Roma, Italy; 6 Institute of Protein Biochemistry, National Research Council, Via P. Castellino 111, 80131 Napoli, Italy

**Keywords:** ALS, Inflammation, IL-1 family, IL-18

## Abstract

**Background:**

Amyotrophic lateral sclerosis (ALS) is a progressive motor neuron disease leading to the death of affected individuals within years. The involvement of inflammation in the pathogenesis of neurodegenerative diseases, including ALS, is increasingly recognized but still not well understood. The aim of this study is to evaluate the levels of inflammation-related IL-1 family cytokines (IL-1β, IL-18, IL-33, IL-37) and their endogenous inhibitors (IL-1Ra, sIL-1R2, IL-18BP, sIL-1R4) in patients with sporadic ALS (sALS),

**Methods:**

Sera were collected from 144 patients (125 patients were characterized by disease form, duration, and disability, using the revised ALS functional rating scale (ALSFRS-R) and from 40 matched controls. Cerebrospinal fluid (CSF) was collected from 54 patients with sALS and 65 patients with other non-infectious non-oncogenic diseases as controls. Cytokines and inhibitors were measured by commercial ELISA.

**Results:**

Among the IL-1 family cytokines tested total IL-18, its endogenous inhibitor IL-18BP, and the active form of the cytokine (free IL-18) were significantly higher in the sALS sera than in controls. No correlation between these soluble mediators and different clinical forms of sALS or the clinical setting of the disease was found. IL-18BP was the only mediator detectable in the CSF of patients.

**Conclusions:**

Among the IL-1 family cytokines, only IL-18 correlates with this disease and may therefore have a pathological role in sALS. The increase of total IL-18 suggests the activation of IL-18-cleaving inflammasome. Whether IL-18 upregulation in circulation of sALS patients is a consequence of inflammation or one of the causes of the pathology still needs to be addressed.

## Background

Amyotrophic lateral sclerosis (ALS) is a fatal neuromuscular disorder characterized by the progressive loss of anterior-lateral horn spinal cord motoneurons, leading to progressive muscular weakness and eventual death of affected individuals [1]. Sporadic ALS (sALS) is the primary form of the disease, affecting 95% of ALS patients, while the remaining 5% of cases have a familial form of the disease.

Several studies have suggested that immune activation and inflammatory mechanisms may have an active role in ALS pathogenesis [2]. It has been reported that patients with sALS exhibit an anomalous monocyte phenotype, with increased expression of CD16 and HLA-class II, which is directly related to the rate of sALS disease progression [3]. Infiltrating macrophages and T cells have been found in the central nervous system (CNS) in human ALS, and immunoglobulin G (IgG) and complement deposition are also evident [2,4-6]. Parallel to cell infiltration, increased levels of pro-inflammatory cytokines such as CCL2 [7] and IL-6 have been reported in both the cerebrospinal fluid (CSF) [8] and sera [9], and increased levels of TNF-α were detected in the blood of these patients [10].

An involvement of IL-1 family cytokines in the pathology of ALS has been suggested by the finding of activated caspase-1 and increased IL-1β both in ALS mouse models and in patients [11-13]. The IL-1 family includes eight cytokines with inflammatory or anti-inflammatory activity (IL-1α, IL-1β, IL-18, IL-33, IL-36α, IL-36β, IL-36γ, IL-37), and three receptor antagonists (IL-1Ra, IL-36Ra, IL-38) [14,15]. Members of the IL-1 receptor (IL-1R) family include four receptor chains and two accessory chains that form four signalling complexes, two orphan receptors, two decoy receptors (IL-1R2, IL-18BP), and two negative regulators [16].

IL-1β and IL-18 are synthesized as biologically inactive precursors and subsequently cleaved by caspase-1 to give rise to mature active cytokines [17]. Also the anti-inflammatory cytokine IL-37 is synthesized as a long protein, which needs caspase-1 both for its nuclear translocation (where it may act as a DNA-binding protein) and for its cleavage and extracellular release as an IL-1-like cytokine [18]. IL-1β exerts its potent inflammatory effect by binding to IL-1R1, and its activity is inhibited by the IL-1R antagonist IL-1Ra, which competes with IL-1β for IL-1R1 binding. IL-1R2 functions as a decoy receptor for IL-1β, capturing the cytokine and preventing its interaction with IL-1R1 [16,19].

IL-18 is cytokine strongly involved in type 1 inflammation, as it induces activation of Th1 cells and production of IFN-γ by Th1 and NK cells [20]. IL-18 is produced by many cell types, including leukocytes, epithelial and endothelial cells, and various cells of the adipose tissue. The inflammation-related effects of IL-18 may vary depending on the microenvironment, and there are reports suggesting that IL-18 could also be involved in type 2 inflammation and Th2 cell activation [21]. Beside inflammatory effects, IL-18 is also involved in regulating energy homeostasis (by controlling food intake, energy expenditure and respiratory exchange) [22,23] and in maintaining the functional integrity of mucosal barrier functions [24]. IL-18 is regulated by the IL-18 binding protein (IL-18BP), a soluble molecule that binds mature IL-18 with high affinity and prevents its interaction with cell surface receptors. Only the fraction of IL-18 that is not bound to IL-18BP is actually free to interact with the membrane receptors on target cells/organs and is biologically active.

Also the anti-inflammatory cytokine IL-37, in its caspase-1-dependent extracellular short form, binds to IL-18 receptors and is inhibited by IL-18BP [14,25]. IL-33 is another member of IL-1 family highly homologous to IL-18 [26]. IL-33 is synthesized as a pro-protein that acts as nuclear factor regulating transcription. Upon inflammasome- and caspase-1-independent cleavage, mature IL-33 is exported extracellularly and acts as a cytokine by binding to its receptor IL-1R4 (previously known as T1/ST2), thereby recruiting and activating leukocytes and inducing Th2-associated cytokines [27]. The soluble form of the receptor, sIL-1R4, is a natural inhibitor of IL-33.

In order to investigate if the IL-1 network and inflammasome hyperactivation have a pathogenic role in sALS by promoting neuroinflammation and contributing to disease progression, we analyzed the levels of IL-1 family molecules (IL-1β, IL-18, IL-33, IL-37) and their endogenous inhibitors (IL-1Ra, sIL-1R2, IL-18BP, sIL-1R4) in serum and CSF from sALS patients.

## Methods

### Subjects

A total of 144 ALS patients were included in the study. Clinical data at the time of blood and CSF drawing were available for 125 patients (Table 1). CSF was collected in 54 patients out of 144.

**Table 1:** Demographic and clinical characteristics of sALS patients

sALS patients (total)	125
Sex (F/M)	69/56
Age (mean and range)	63.28 (32–82)
Disease duration (in months; mean and range)	11.32 (1–48)
ALSFRS-R (mean and range)	44 (35–48)
Bulbar (n/total)	22/125
Upper limb (n/total)	45/125
Lower limb (n/total)	58/125

sALS, sporadic amyotrophic lateral sclerosis, ALSFRS-R, revised ALS functional rating scale. Bulbar, upper limb, lower limb is a clinical classification based on involved body areas at the onset of disease. Cases with only bulbar signs or symptoms for the first three months from onset, with spinal symptoms or signs occurring later, are considered bulbar onset ALS. Cases in which any spinal symptoms or signs appeared in the first three months are considered spinal onset ALS (either upper or lower limb).

To evaluate overall patient functional status, the Revised ALS Functional Rating Scale (ALSFRS-R, scored 0–48) was used [28]. All patients had ALS with a range of ALSFRS-R scores of between 35 and 48. Disease onset had occurred in patients between 1 and 96 months before the time of sampling. A total of 40 healthy serum donors (30 females and 10 males, mean age 51 ± 19 years), and 65 CSF donors (37 females and 28 males, mean age 56 ± 20 years) with different kinds of non-infectious non-inflammatory pathologies were used as controls. Written informed consent was obtained from all subjects. The study was approved by the local Ethics Committees.

### Cytokine assay in serum and CSF

Cytokine levels in serum and CSF were measured by ELISA with commercially available assays. The ELISA kit for human IL-18 was obtained from MBL (Woburn, Massachusetts, United States), kits for hIL-18BPa, hIL-1β, hIL-1Ra, hsIL-1R2, hIL-33, and hsIL-1R4 were purchased from R&D Systems Inc. (Minneapolis, New Mexico, United States), while the ELISA kit for hIL-37 was provided by AdipoGen, Inc. (Seoul, South Korea). The IL-18 kit from MBL could detect total IL-18 (either free or bound to IL-18BP), as described previously [29]. Results obtained with the IL-18BPa ELISA were adjusted by considering the different molecular weight of the standard (a chimeric protein), as already described [30]. The concentration of free IL-18 (biologically active cytokine not bound by its inhibitor) was calculated as previously described [30], by applying the law of mass action to the measured concentrations of IL-18 and IL-18BP, considering that the interaction between the two factors is 1:1 and that the K_d_ of their binding is 400 pM [31].

### Statistical analysis

Analysis of the data was performed using the Mann–Whitney *U*-test. Spearman’s rank correlation analysis was used to correlate disease activity score and serum levels of cytokines (total IL-18, IL-18BP and free IL-18). A *P* value of less than 0.05 was considered statistically significant.

## Results

**Figure 1:**
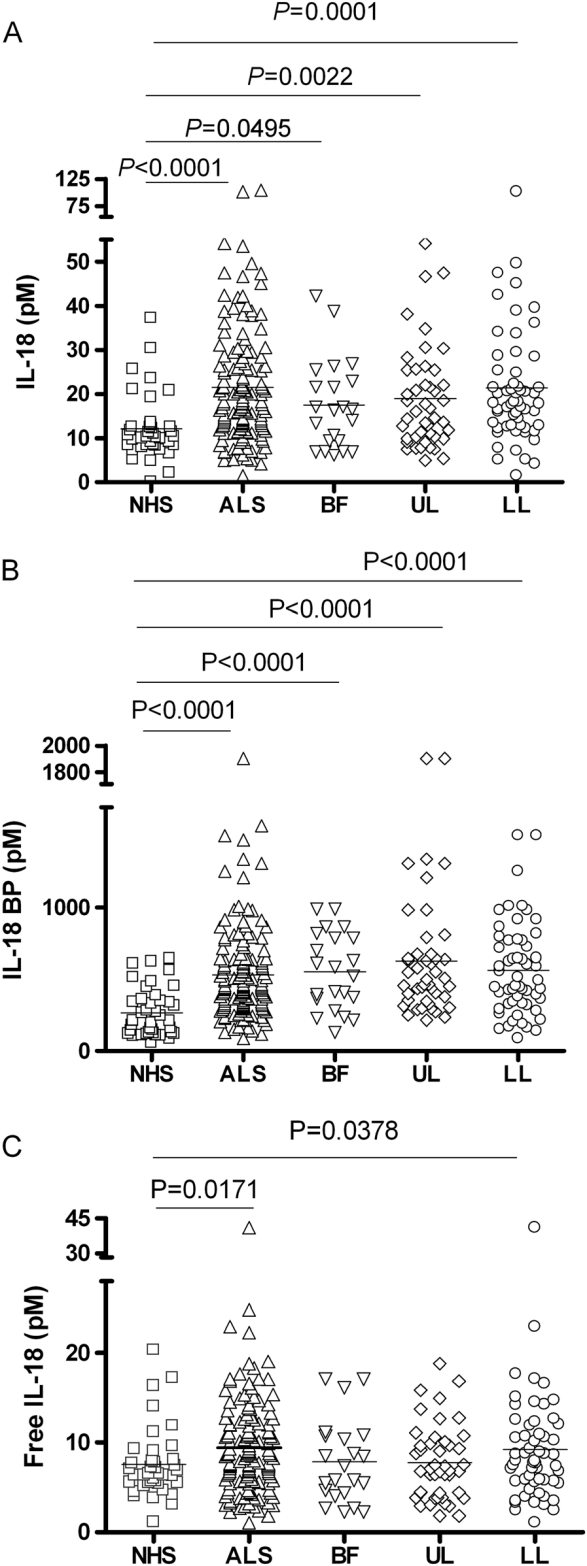
**Levels of IL-18, IL-18BP and free IL-18 in serum of ALS patients.** Levels of IL-18 **(A)** and of IL-18BP **(B)** were measured by ELISA in sera from sporadic ALS patients (sALS) in comparison to NHS. Patients were also grouped depending on the form of the disease. Based on the law of mass action, the concentration of free IL-18 (not bound and inhibited by IL-18BP) was calculated from total IL-18 and total IL-18BP concentrations [30,31]**(C)**. Statistical significance was calculated using the Mann–Whitney *U*-test. ALS, amyotrophic lateral sclerosis; BF, bulbar form; LL, lower limbs; NHS, normal healthy subjects; UL, upper limbs.

IL-1β was undetectable in the serum of sALS patients, with the exception of one ALS patient that had measurable circulating IL-1β (Table 2). At variance with IL-1, IL-18 was measurable in sera and its levels were significantly increased in sALS compared to normal healthy subjects (NHS) (*P* <0.0001, Figure 1A). The soluble inhibitor of IL-18, IL-18BP, was similarly increased in the serum of sALS patients as compared to NHS (*P* <0.0001, Figure 1B). Despite the increase in IL-18BP, serum levels of free IL-18 were still significantly higher in ALS patients than in NHS controls (*P* = 0.0171, Figure 1C).

**Table 2:** IL-1 family cytokine and receptor levels in CSF and serum of sALS patients

**Cytokine/Receptor**	**CSF**		**Serum**	
	**ALS**	**Controls**	**ALS**	**Controls**
**IL-1β**	<7.8	<7.8	<7.8 (n = 1; 47.2)	<7.8 (n = 1; 26.6)
**IL-1Ra**	<78.1	<78.1 (n = 1; 101.7)	<78.1 (n = 1; 547.4)	<78.1
**sIL-1R2**	<312.5	<312.5 (n = 1; 325.7)	5673.4 ± 2771.5	5575.0 ± 1359.5
**IL-33**	<30.0 (n = 3; 72.9 ± 32.3)	<30.0 (n = 3; 54.8 ± 27.1)	625.4 ± 1570.5	536.3 ± 1166.3
**sIL-1R4**	<62.5 (n = 4; 85.1 ± 13.2)	<62.5(n = 2; 85.5 ± 3.9)	72.4 ± 16.4	79.9 ± 28.3
**IL-37**	<46.8	<46.8	<46.8	<46.8 (n = 1; 74.7)
**IL-36**	NT	NT	888.3 ± 729.3	1531.7 ± 1843.2

Values are given as mean pg/ml ± SD rather than in nM since the MW of the mature forms of the cytokines and of the natural soluble receptors are not yet precisely defined.

CSF, Cerebrospinal fluid; n, Number of samples above the detection limit; NT, Not tested; sALS, sporadic amyotrophic lateral sclerosis.

IL-1β and IL-18 were not detectable in the CSF of sALS patients or healthy and disease controls (patients with non-infectious non-inflammatory neurological diseases) (Table 2). IL-18BP was detected in the CSF of sALS patients (mean ± SD: 99 ± 67 pM) as well as in disease controls (91 ± 69 pM) and in healthy controls (156 ± 116 pM), but these differences were not statistically significant. The presence of two inhibitors of IL-1β in serum and CSF was evaluated. IL-1Ra was not detectable in NHS sera; IL-1Ra was only detected (547 pg/ml) in the serum of the one sALS patient who had measurable IL-1β. In CSF, IL-1Ra was found only in one control (102 pg/ml), without obvious correlation with disease state. The other IL-1 inhibitor, the soluble form of the IL-1R2 (sIL-1R2), was present in sera of sALS patients in amounts similar to controls (Table 2). In CSF, sIL-1R2 was detectable in one healthy control only (326 pg/ml). IL-33 and sIL-1R4 levels were comparable in sALS and normal sera. In CSF, the cytokine and its soluble receptor were undetectable, with the exception of a few patients and controls (Table 2).

Another important inhibitor of inflammation that has been recently described is IL-37, a cytokine of the IL-1 family structurally very similar to IL-1 and IL-18, and allegedly able to bind to IL-18BP [32]. The most abundant isoform of the cytokine, IL-37b, was undetectable in the CSF and serum of both sALS patients and controls (Table 2). We then analyzed the relationship between cytokine levels and clinical parameters of the disease. Free serum IL-18 (as well as total IL-18 and IL-18BP) did not significantly correlate with the ALSFRS-R score, nor with the time from diagnosis (data not shown). When patients were subdivided according to clinical presentation in bulbar, upper limbs and lower limbs form, total IL-18 and IL-18BP serum levels were significantly higher in each subgroup compared to NHS (Figure 1A and B). Free IL-18 was significantly increased in lower limbs form with respect to controls (Figure 1C).

## Discussion

The data collected in the current study shows that, among the cytokines of the IL-1 family tested, only IL-18 and IL-18BP are increased in the serum of sALS patients. Measuring both the active cytokine and its inhibitor in serum is required in order to correctly evaluate the net biological effects, as only free IL-18, not captured by its inhibitor, is able to exert its biological effects [31]. It should be noted that an excess of over 100-fold inhibitor is not sufficient for full inhibition of the active cytokine because inhibition efficiency depends both on the kinetics of association and dissociation between the two molecules, and on the actual concentration of IL-18 and IL-18BP in serum rather than on their ratio. Thus, despite the overproduction of IL-18BP in these patients, it is nevertheless insufficient for controlling the excessive production of IL-18. As a consequence, free active IL-18 is significantly higher in sALS patients than in healthy individuals. Whether this increase in the active cytokine levels may have a pathological significance is not known. Increased IL-18 levels are associated with a variety of diseases and, in general, to ongoing inflammation. High levels of circulating IL-18 are found also in mild inflammatory conditions such as in the elderly or in obese individuals [33-35]. In addition, IL-18 can have either protective or detrimental effects depending on the tissue microenvironmental conditions, for instance in the regulation of allergic reactions [36] or in the protection versus destruction of the mucosal tissue [24,37,38].

High concentrations of circulating IL-18 may suggest the involvement of inflammasome-dependent inflammation in the disease. The inflammasome, a cytosolic molecular complex that controls the activation of caspase-1 [39], is a key component of the inflammatory machinery. Once activated, caspase-1 processes pro-IL-1β and pro-IL-18, two inflammatory cytokines that require cleavage in order to attain their active forms. The levels of IL-18 can provide information of inflammasome activation and local IL-1β production, since circulating IL-1β is detectable only in extreme or fatal situations of shock due to its potent bioactivity. On the other hand, IL-18, which is much less dangerous than IL-1β as it has not vasoactive effects, can be found at detectable levels in the serum of normal subjects and it increases in pathological and inflammatory conditions, thus acting as a sign of an ongoing inflammasome-related inflammation.

In the SOD1-G93A mouse model of ALS, the disease is caused by a mutation in superoxide dismutase 1 (SOD1) that provokes enhanced inflammation and the production of a broad spectrum of inflammatory cytokines [40-42]. Active caspase-1 is a marker of inflammation in the CNS of SOD1-G93A mice [11,12]. Indeed, microglia and macrophages from these mice show high levels of caspase-1 activation and concomitant increase in the secretion of mature IL-1β [1]. SOD1 mutations are the most common genetic cause of ALS in humans (accounting for 20% of familial ALS cases) [43], although in about 95% of ALS patients no genetic cause can be identified (sALS patients) [44].

In mouse models and in familial ALS patients, activation of caspase-1 has been detected in spinal cords and, consequently, mature IL-1β levels were higher in spinal cord samples [11,12]. Thus, the present study suggests that caspase-1 activation may also be involved in the pathogenesis of sALS, in which the SOD1 mutation is not present.

According to our data, IL-18 and IL-1β are not detectable in CSF, suggesting a predominantly tissue-restricted activity of the inflammatory cytokines. On the contrary, IL-18BP is present in CSF, but it does not seems to be associated to the disease since its levels are comparable between healthy or disease controls and sALS patients. Another ligand of IL-18BP that is also able to bind the alpha chain of the IL-18 receptor is IL-37, a cytokine that down-regulates inflammation by inhibiting the gene expression of a range of inflammatory cytokines [25,45]. IL-37 increases in response to inflammatory stimulation, probably acting as a self-regulator of the defensive inflammatory reaction [25,45], and its presence has been observed in several inflammatory disease conditions (for example in the mucosa of patients with Crohn’s disease) [46]. IL-37 was not detected in the serum or CSF from sALS patients or controls. In the absence of measurable levels of known IL-18BP ligands in the CSF, the high constitutive levels of IL-18BP may suggest the physiological need to maintain an anti-inflammatory microenvironment in CNS.

Previous studies suggest that other inflammatory cytokines may play a role in ALS. Increased levels of IL-6 have been observed in CSF and serum of ALS patients [8,9], and TNF-α was elevated in blood of patients [10]. The chemokine CCL2 (chemoattractant and regulator of blood–brain barrier permeability [47]) was also increased in the serum and CSF of patients with ALS [48]. On the whole, these data, and our finding of raised serum levels of free IL-18, support the hypothesis that inflammatory mechanisms may have an active role in the disease process of ALS. In addition, our data suggests that in the sporadic form of the disease inflammasome-dependent caspase-1 activation may be involved, although the mechanisms of inflammasome activation would obviously be different than those in familial ALS. Despite the fact that IL-1β is not detectable in the circulation, it is likely that a local increase of this cytokine may occur and take part in the disease development, whereas the increased levels of circulating IL-18 may be symptomatic of an ongoing caspase-1-dependent inflammatory reaction but not directly involved in the pathology. In parallel to the notion that caspase-1 is activated in sALS, the absence of circulating IL-1Ra and the unchanged levels of sIL-1R2, the two inhibitors of IL-1β activity, suggest that IL-1β would exclusively exert its pathological effects at the local level. Overall, these results suggest that anti-inflammatory approaches based on IL-1β inhibition may be beneficial in treating sALS.

## Conclusions

In the sALS patients we studied, IL-18 levels were significantly increased, but did not correlate with disease severity. This suggests a role of the cytokine in the pathological mechanisms underlying the disease, rather than in disease progression. Published data from Meissner *et al.* in the SOD1-G93A mouse model [13] showed that deficiency in caspase-1 IL-1β or IL-18 did not modify disease onset, thus suggesting that caspase-1-dependent inflammation does not initiate the disease. On the other hand, both caspase-1 and IL-1β (but not IL-18) deficiency extended the lifespan of ALS mice, suggesting that IL-1β-dependent inflammation is involved in the disease process. Thus, circulating IL-18 appears to be a marker of neuroinflammation, indicating caspase-1 activation and local IL-1β effects, whereas it does not represent a useful biomarker in the follow-up of sALS patients.

It is presently unclear if inflammation has a role in initiating neuronal degeneration in ALS, as demonstrated for other neurodegenerative diseases, or it rather is the result of neurodegeneration that then contributes to exacerbate the disease [49-51]. In any case, the experimental evidence supports the hypothesis that deregulated inflammatory reactions, in addition to genetic and environmental factors, could be a key mechanism in the development of sALS, if not its initiation. This further suggests that anti-inflammatory treatments, including inhibition of IL-1β, could be beneficial in ALS patients.

## Abbreviations

ALS: Amyotrophic lateral sclerosis; ALSFRS-R: Revised ALS Functional Rating Scale; BF: bulbar form; CNS: Central nervous system; CSF: Cerebrospinal fluid; IgG: Immunoglobulin G; IL: Interleukin; IL-18BP: IL-18 binding protein; IL-1R: IL-1 receptor; LL: Lower limbs; NHS: Normal healthy subjects; sALS: Sporadic ALS; sIL-1R2: Soluble IL-1R2; SOD1: Superoxide dismutase 1; UL: Upper limbs.

## Competing interests

The authors declare that they have no competing interests.

## Authors’ contribution

DB conceived the study. PM and DB designed and coordinated the study’s experimental activities. PB, BB, CC and GS collected and characterized the ALS samples. PI, IP and PG performed the experiments. PM performed the statistical analysis. PI, IP, PM and DB wrote the manuscript. All authors read and approved the manuscript.
